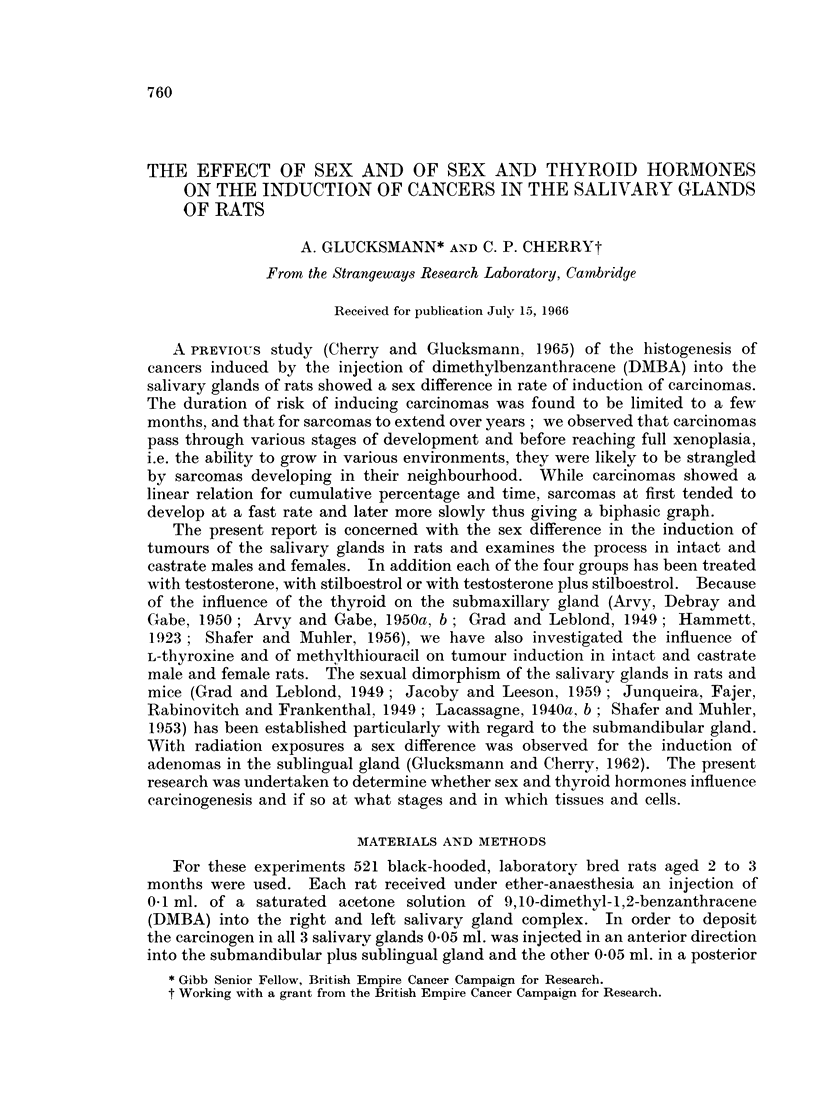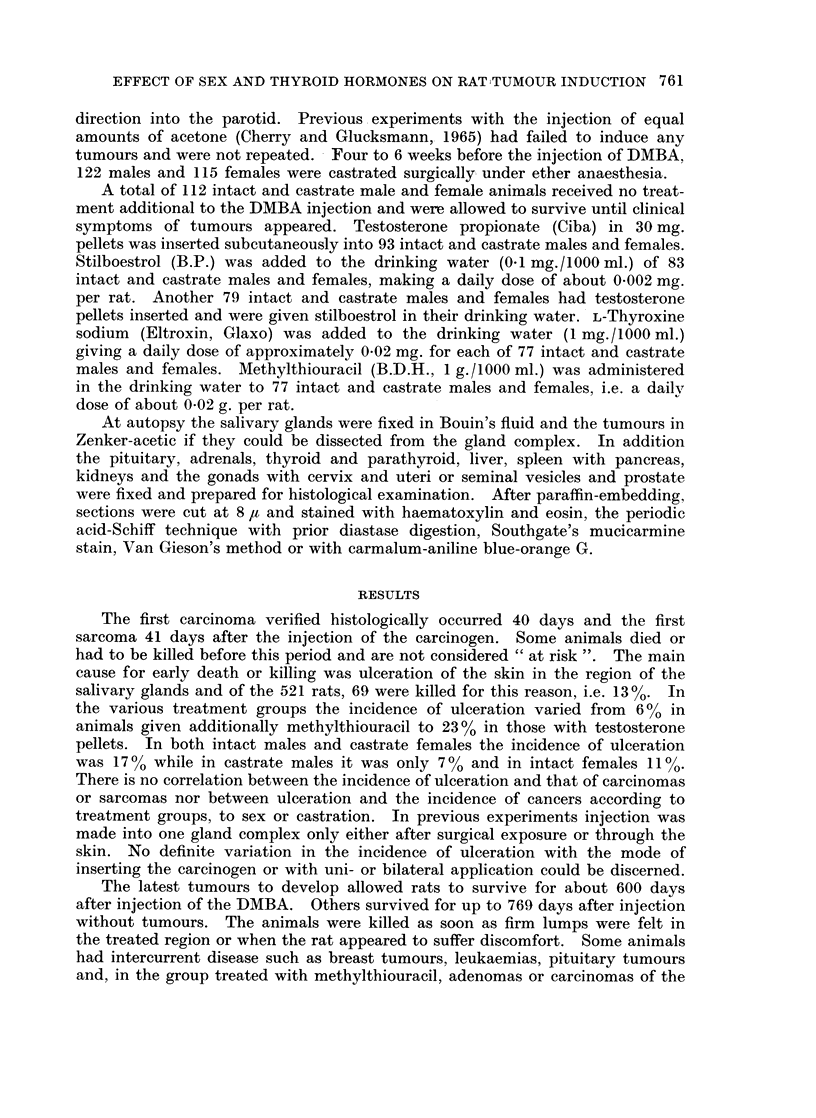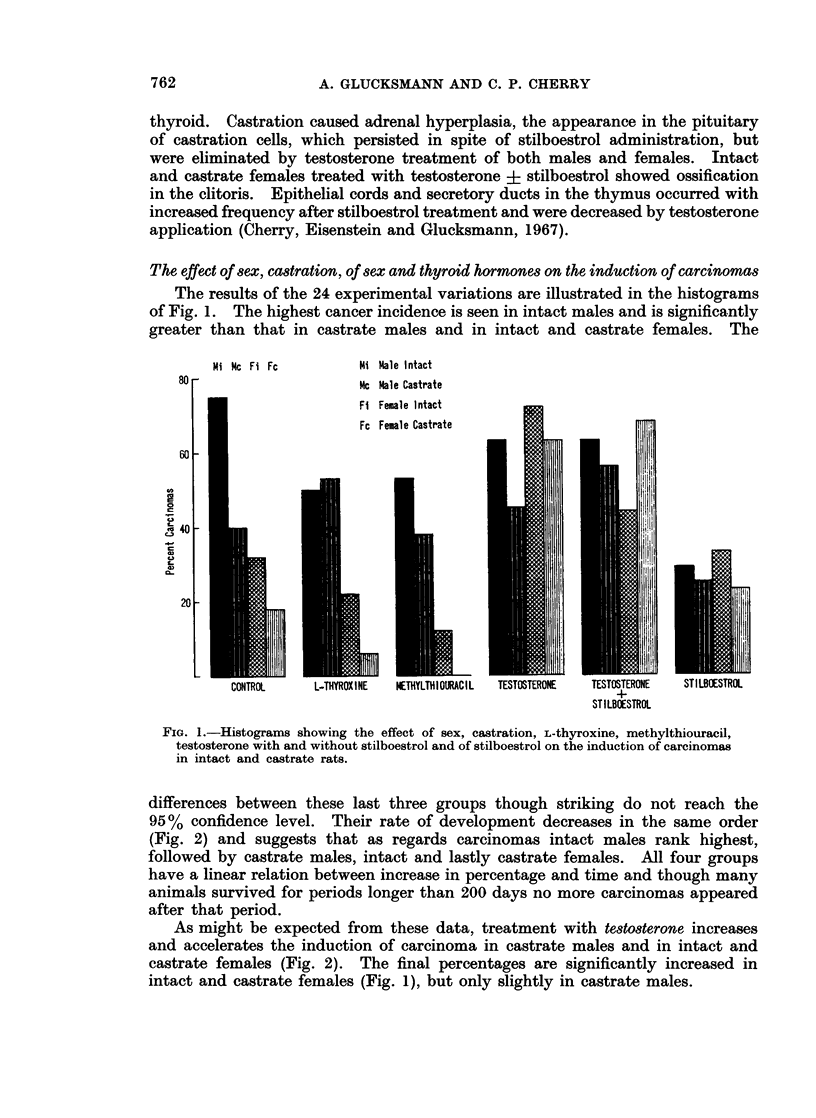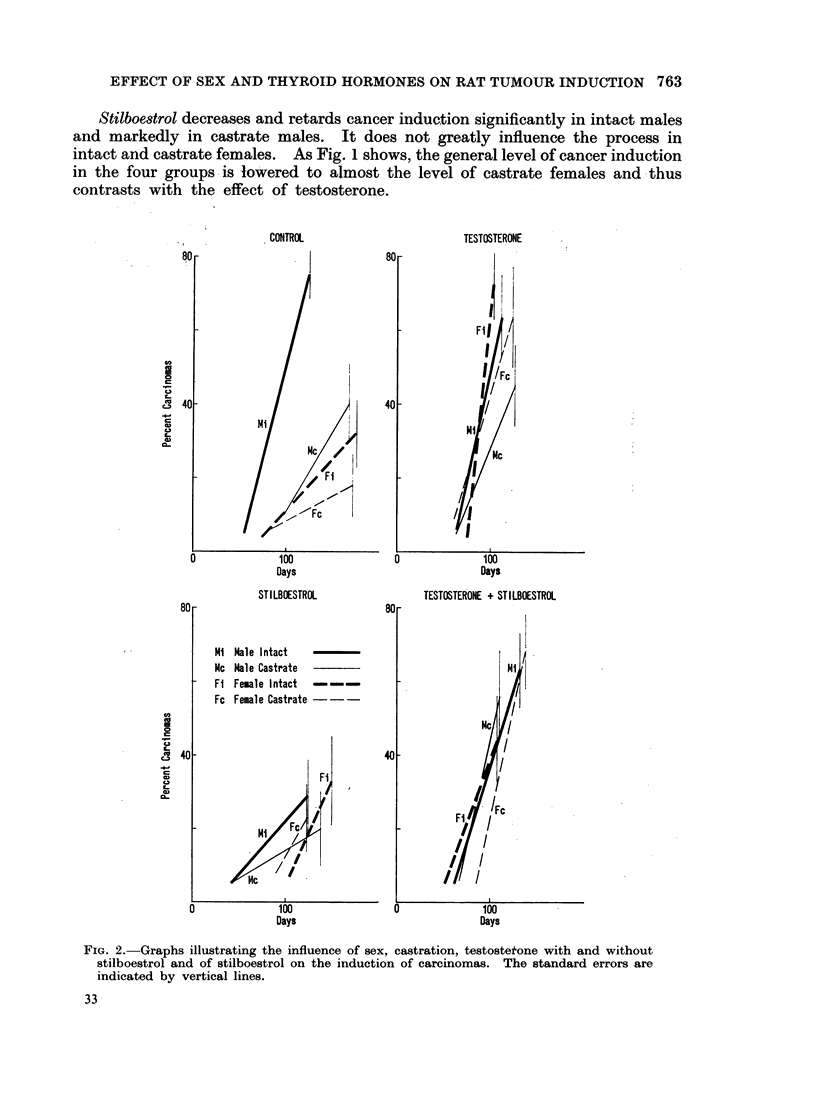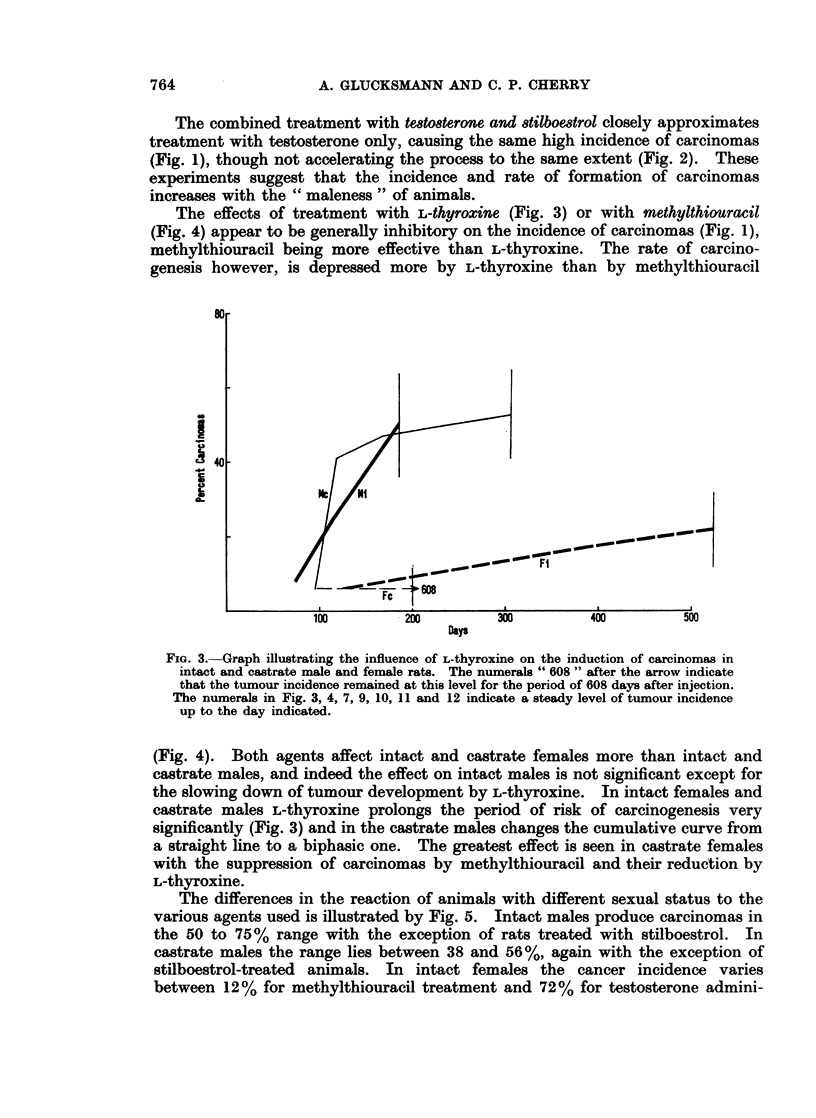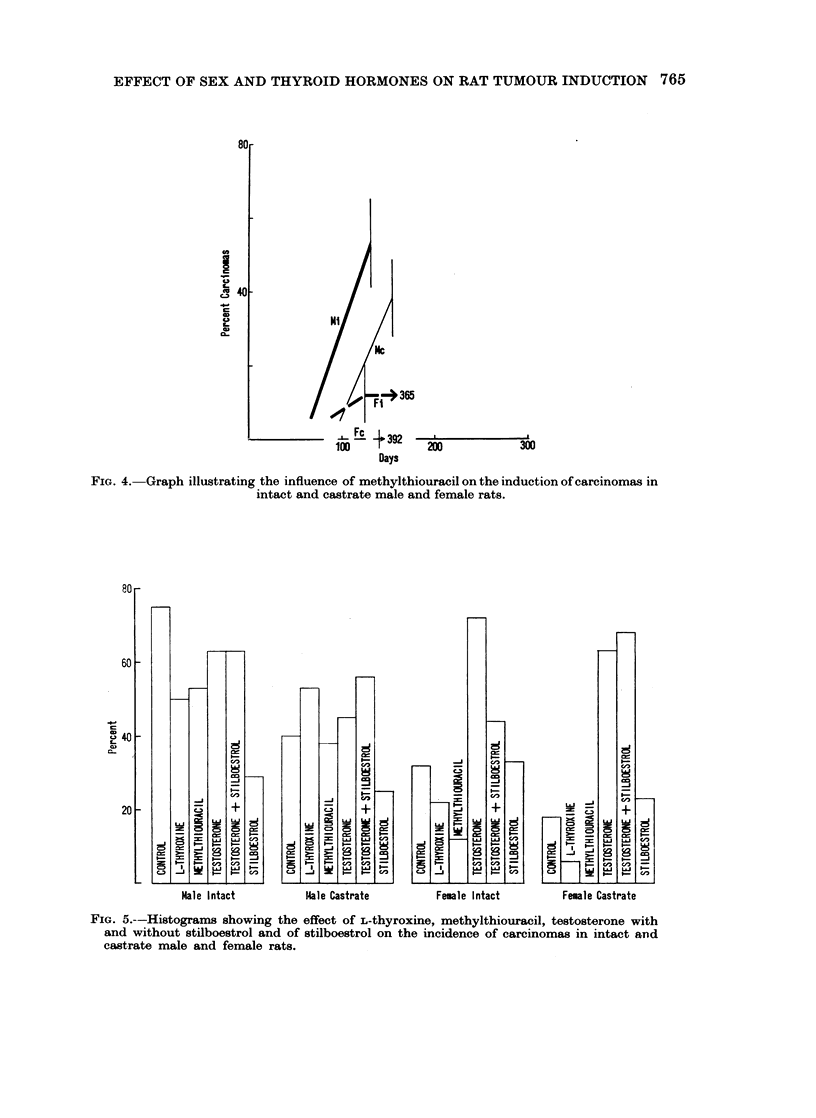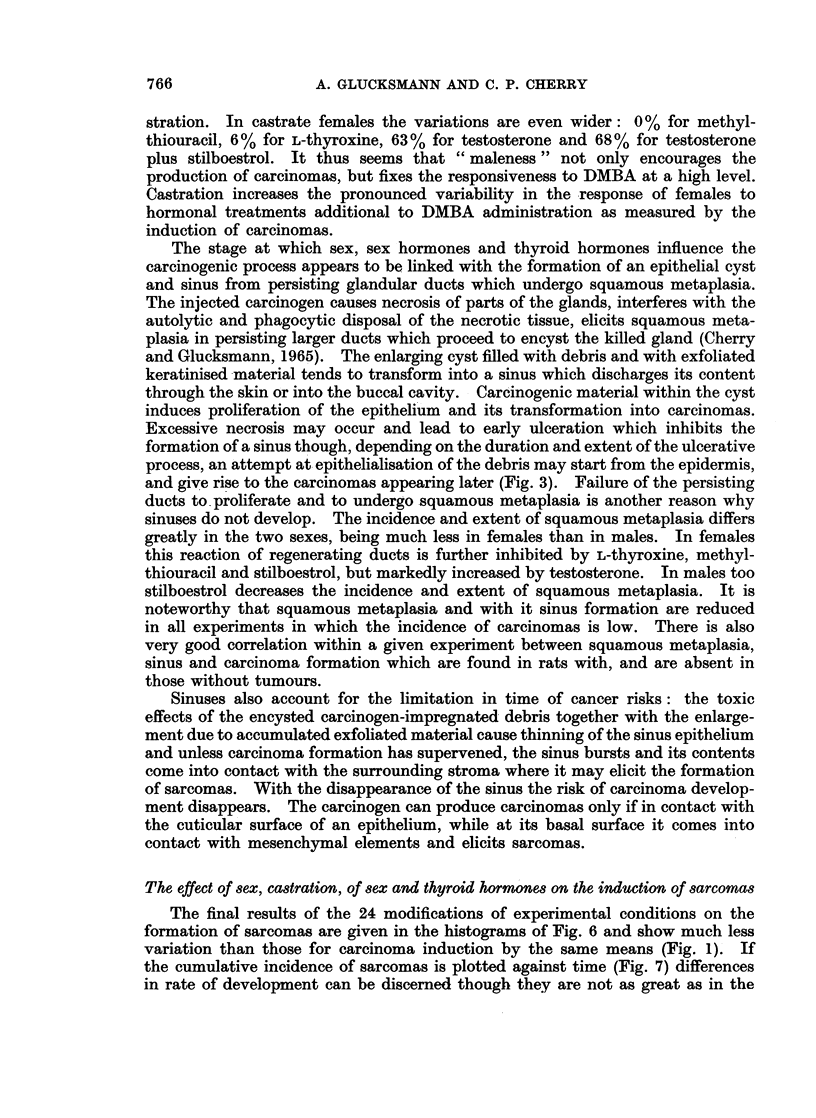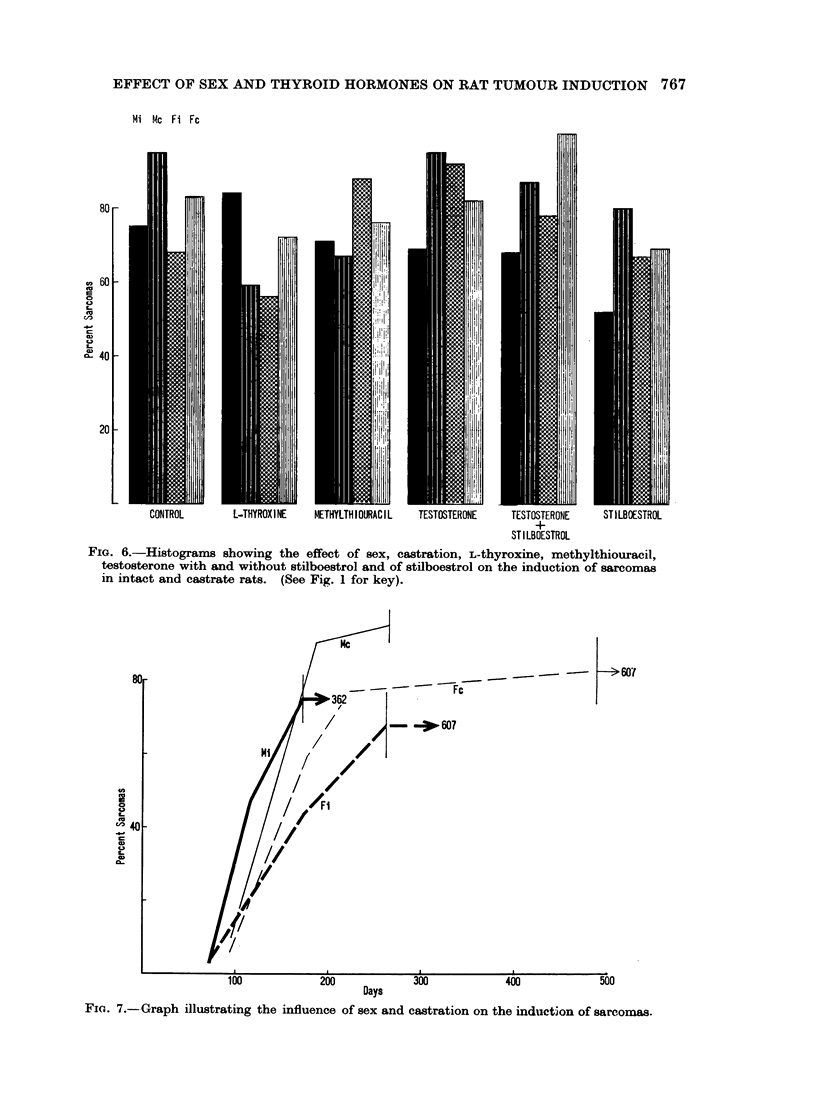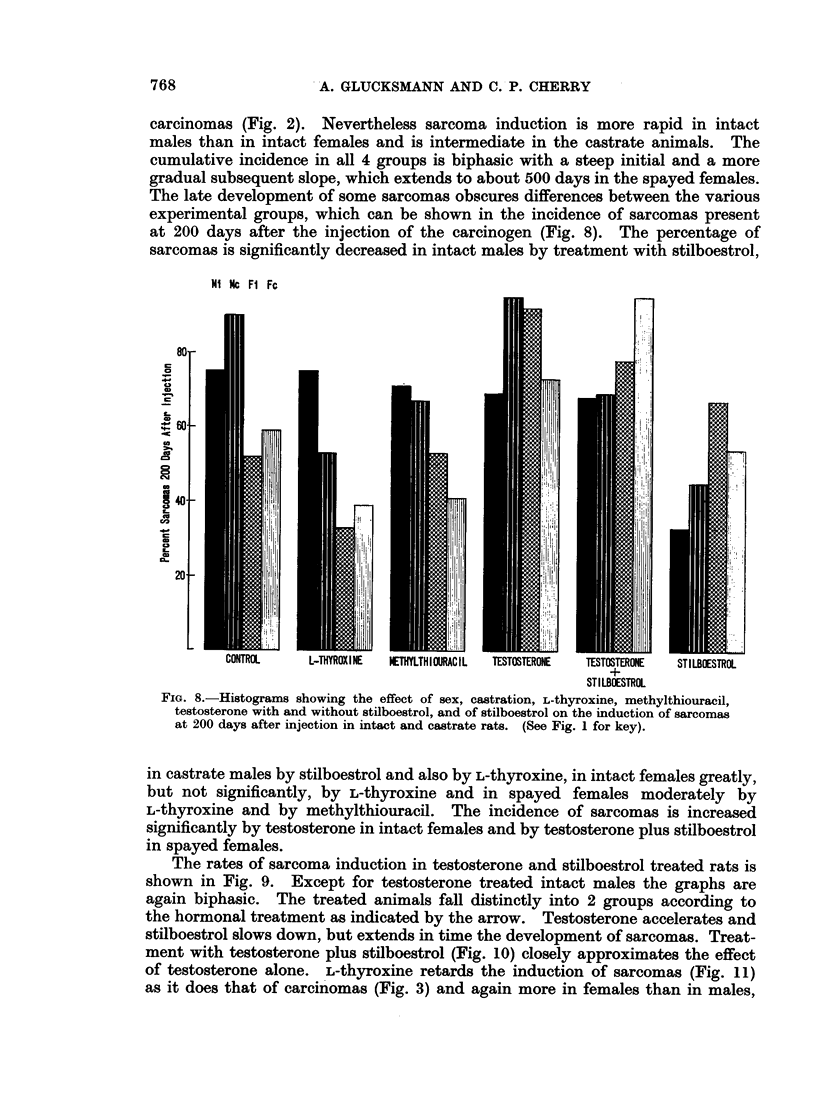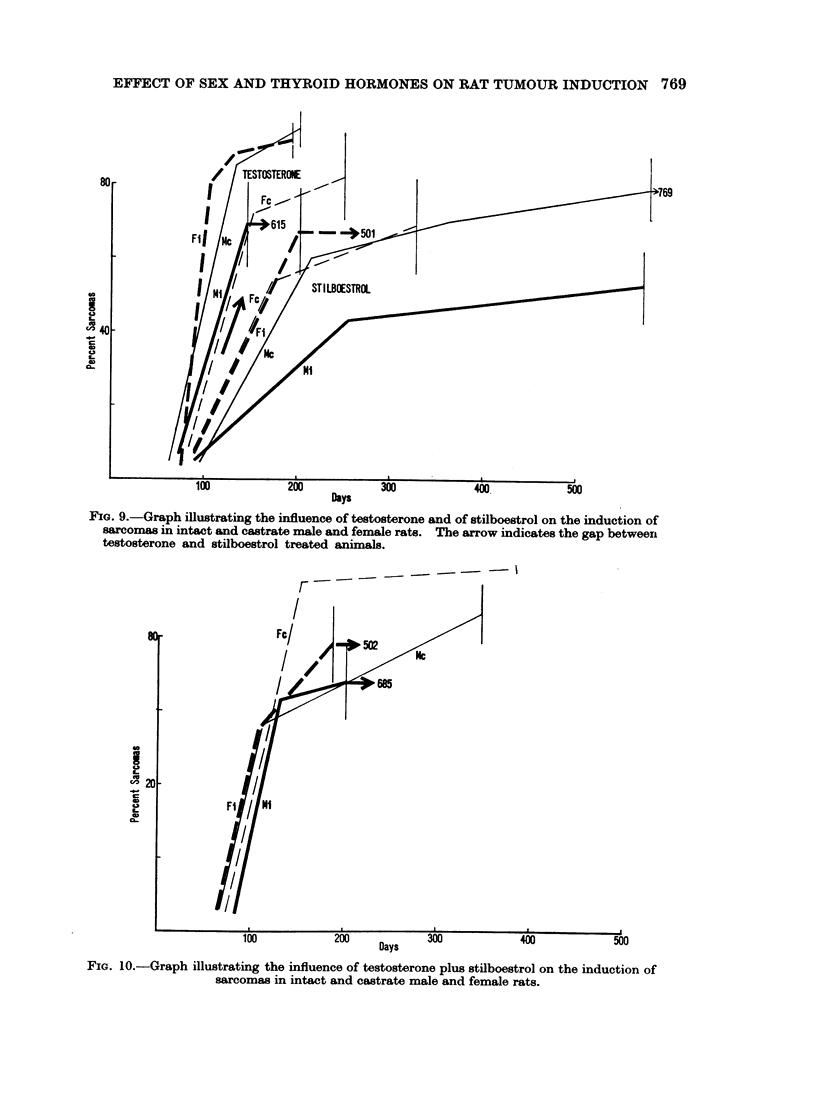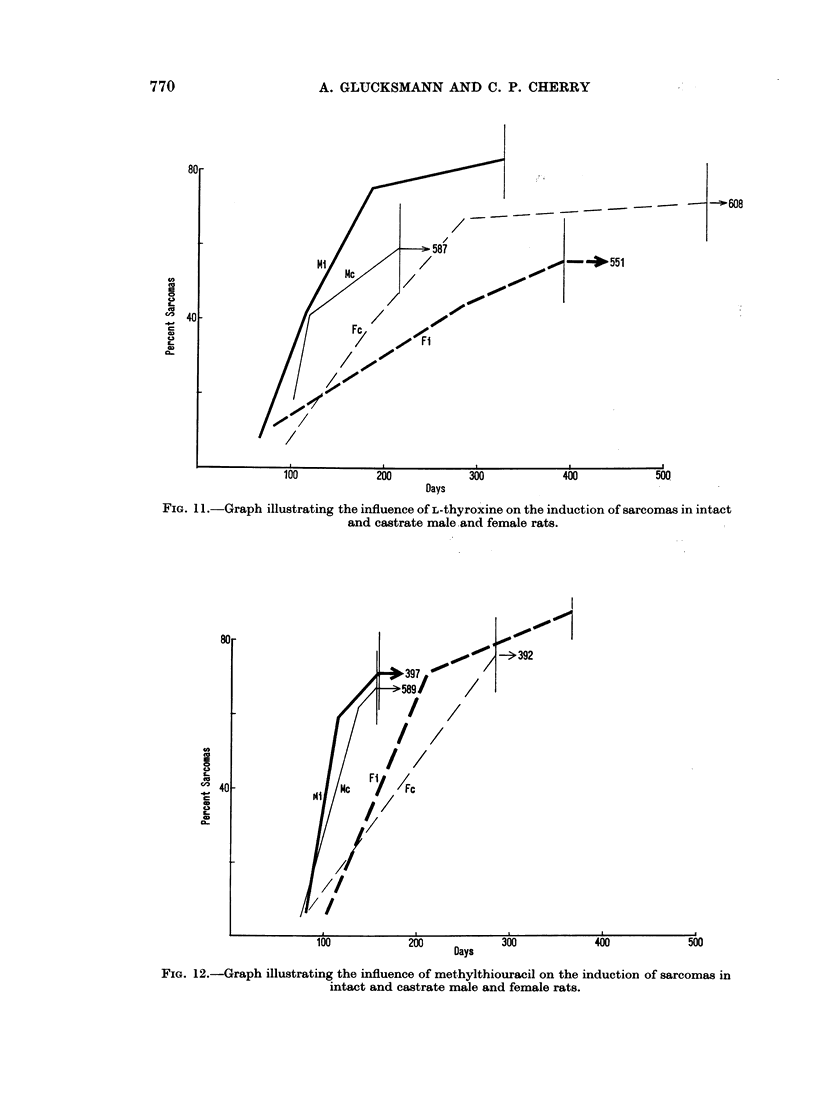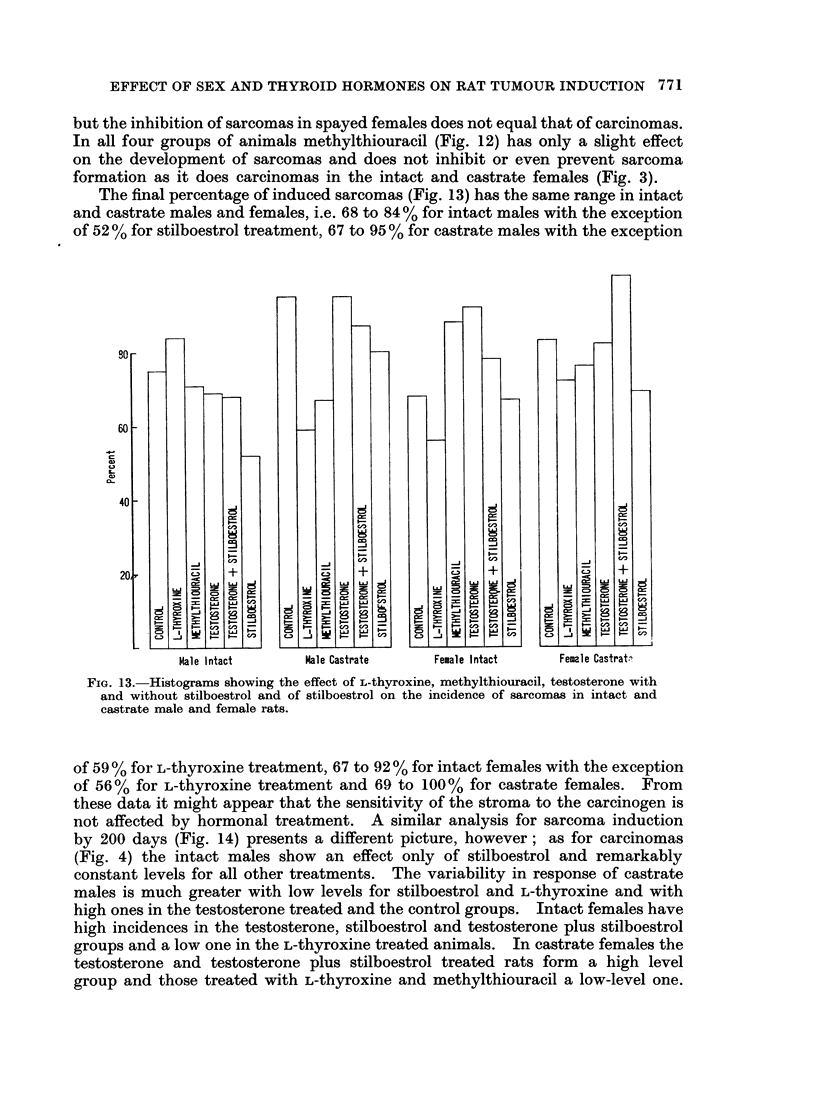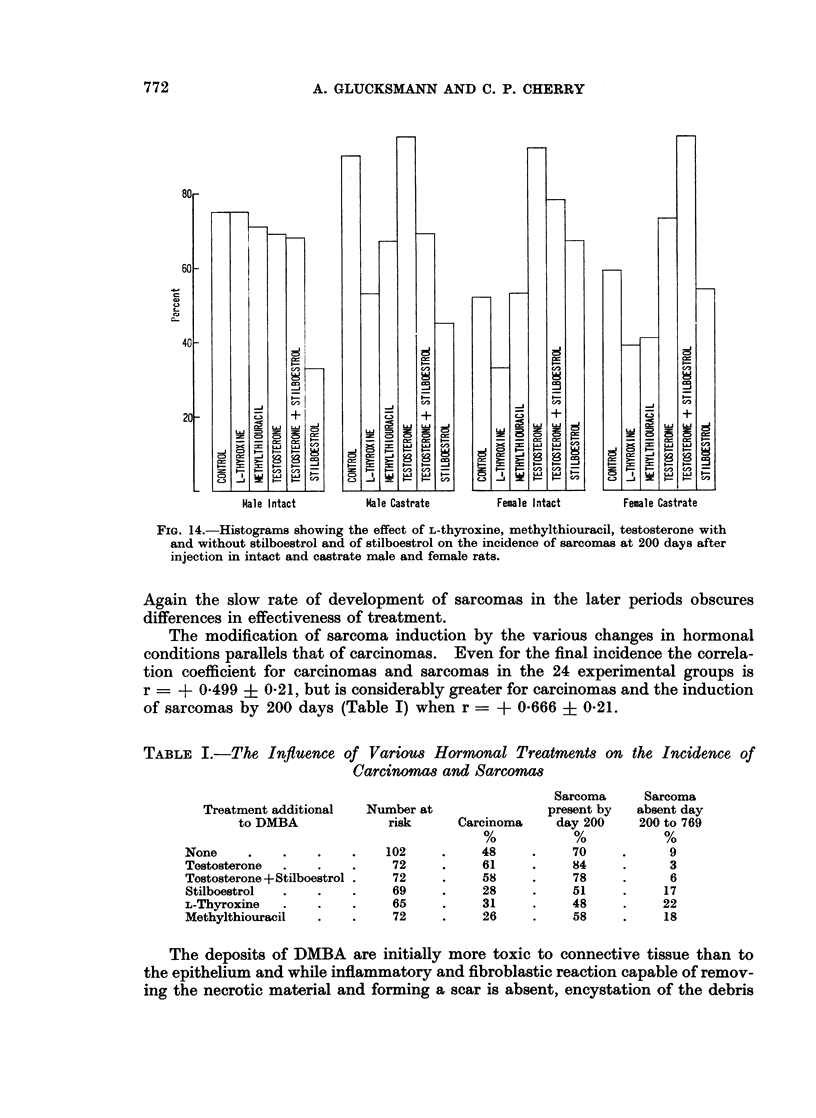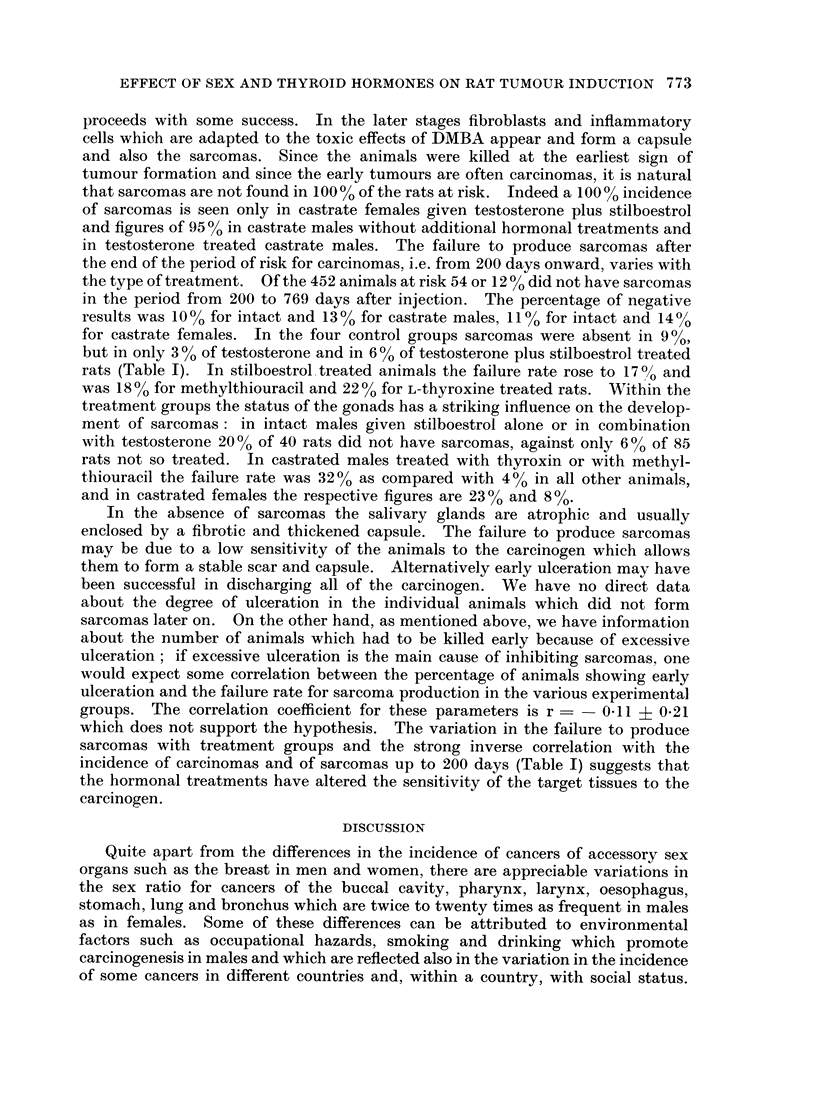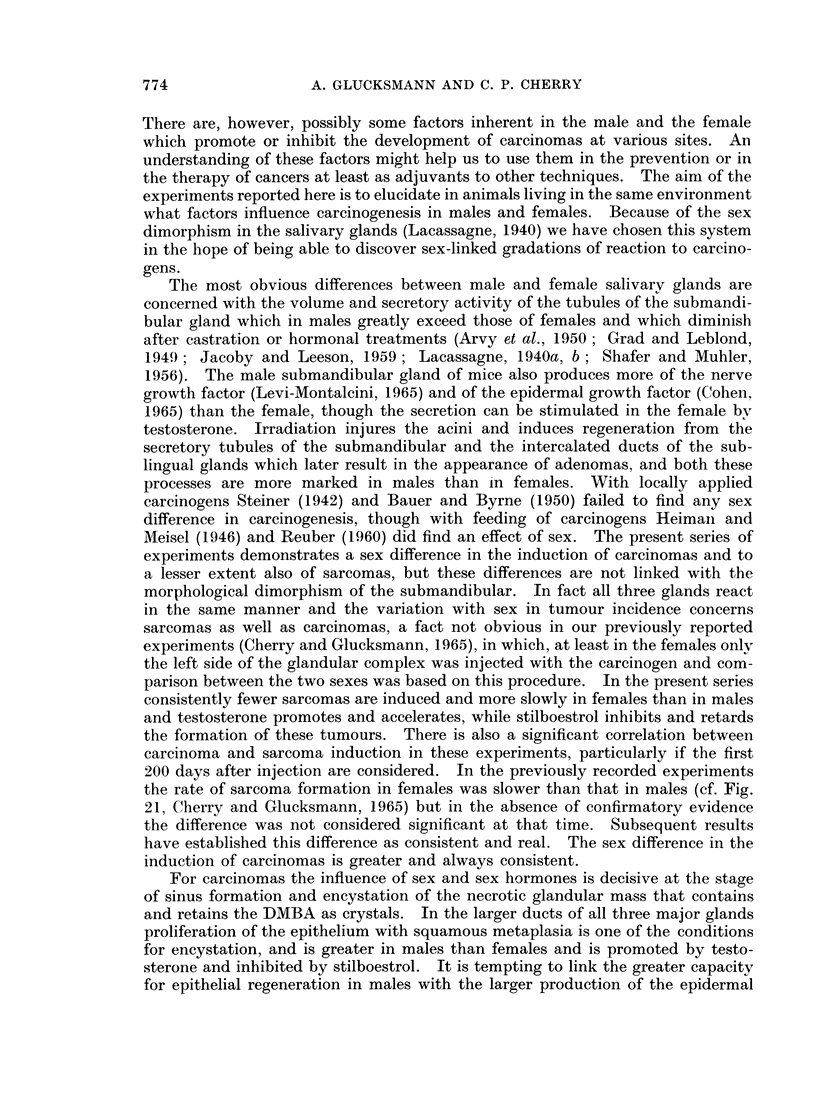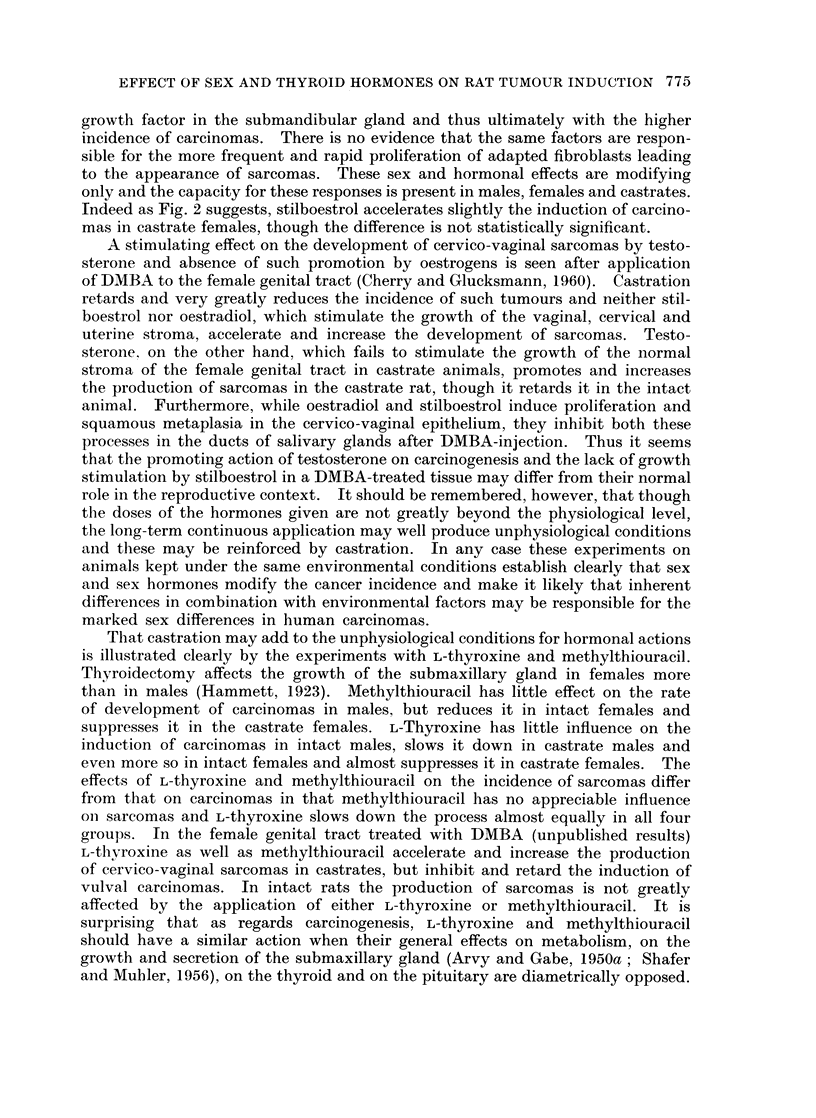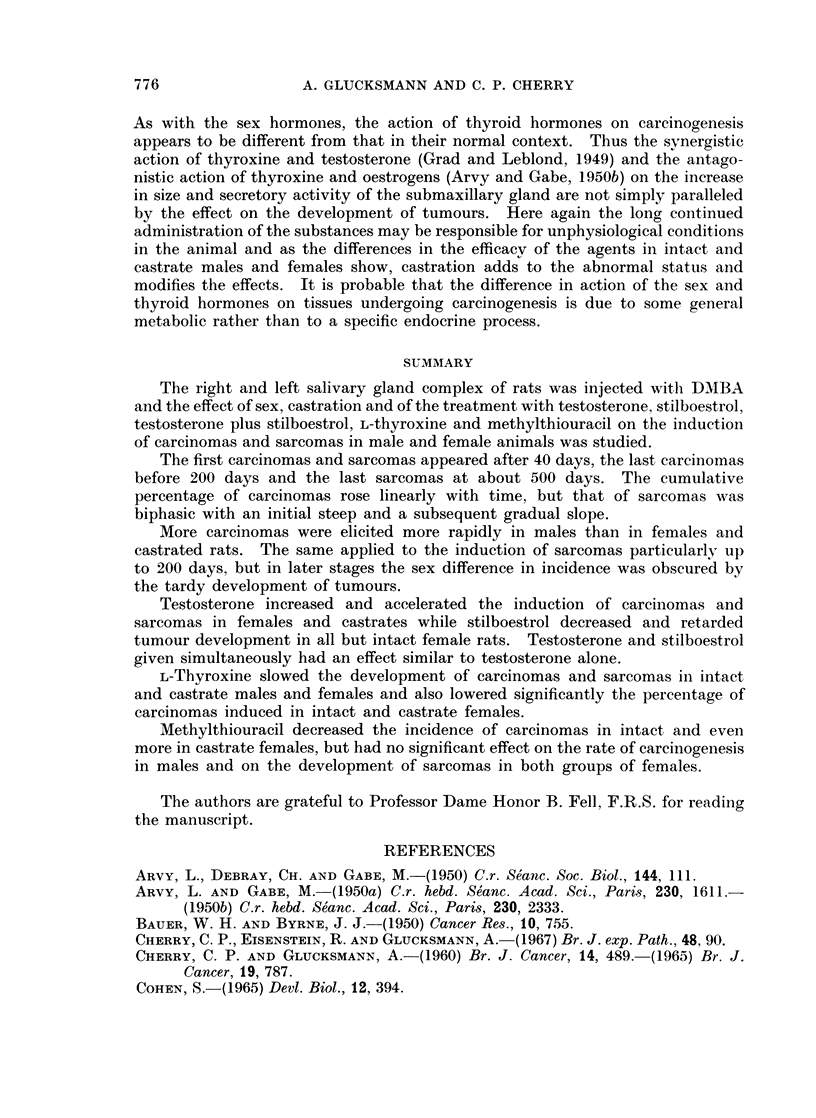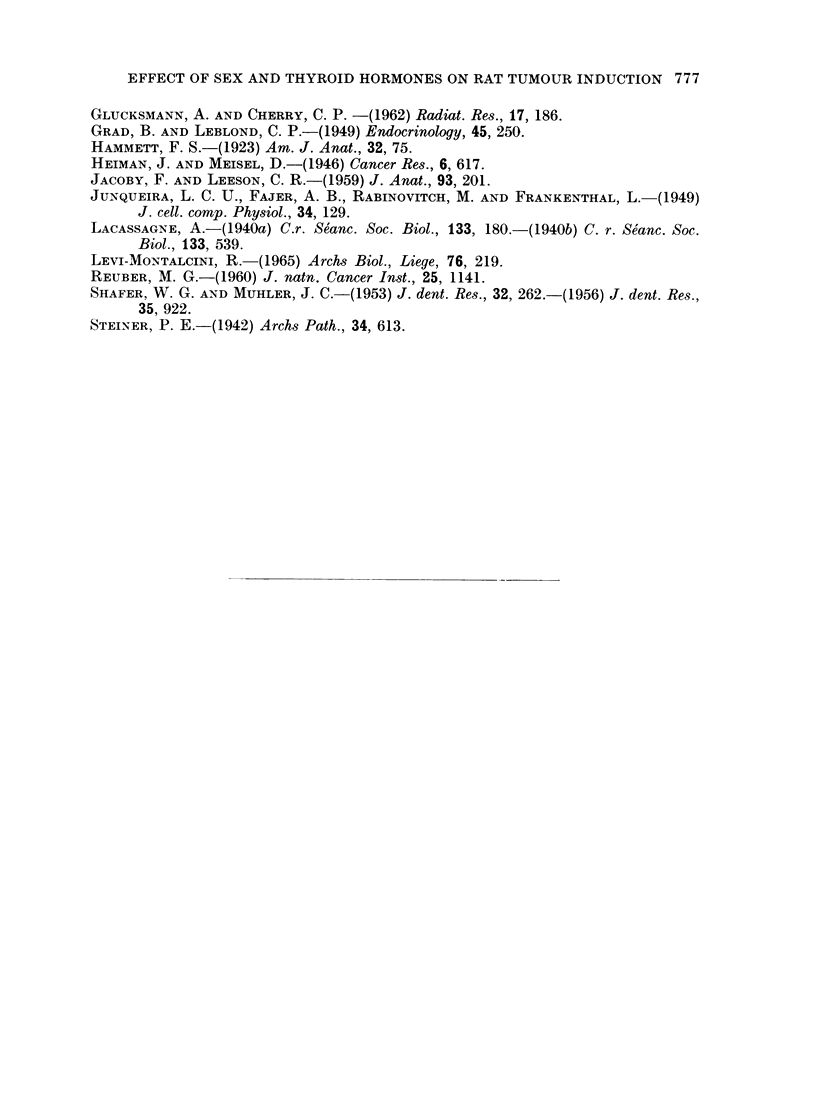# The effect of sex and of sex and thyroid hormones on the induction of cancers in the salivary glands of rats.

**DOI:** 10.1038/bjc.1966.88

**Published:** 1966-12

**Authors:** A. Glucksman, C. P. Cherry


### THE EFFECT OF SEX AND OF SEX AND THYROID HORMONES ON THE INDUCTION OF CANCERS IN THE SALIVARY GLANDS OF RATS

A. GLUCKSMANN* AND C. P. CHERRY†

*From the Strangeways Research Laboratory, Cambridge*

Received for publication July 15, 1966

A PREVIOUS study (Cherry and Glucksmann, 1965) of the histogenesis of cancers induced by the injection of dimethylbenzanthracene (DMBA) into the salivary glands of rats showed a sex difference in rate of induction of carcinomas. The duration of risk of inducing carcinomas was found to be limited to a few months, and that for sarcomas to extend over years ; we observed that carcinomas pass through various stages of development and before reaching full xenoplasia, i.e. the ability to grow in various environments, they were likely to be strangled by sarcomas developing in their neighbourhood. While carcinomas showed a linear relation for cumulative percentage and time, sarcomas at first tended to develop at a fast rate and later more slowly thus giving a biphasic graph.

The present report is concerned with the sex difference in the induction of tumours of the salivary glands in rats and examines the process in intact and castrate males and females. In addition each of the four groups has been treated with testosterone, with stilboestrol or with testosterone plus stilboestrol. Because of the influence of the thyroid on the submaxillary gland (Arvy, Debray and Gabe, 1950 ; Arvy and Gabe, 1950*a*, *b* ; Grad and Leblond, 1949 ; Hammett, 1923 ; Shafer and Muhler, 1956), we have also investigated the influence of L-thyroxine and of methylthiouracil on tumour induction in intact and castrate male and female rats. The sexual dimorphism of the salivary glands in rats and mice (Grad and Leblond, 1949 ; Jacoby and Leeson, 1959 ; Junqueira, Fajer, Rabinovitch and Frankenthal, 1949 ; Lacassagne, 1940*a*, *b* ; Shafer and Muhler, 1953) has been established particularly with regard to the submandibular gland. With radiation exposures a sex difference was observed for the induction of adenomas in the sublingual gland (Glucksmann and Cherry, 1962). The present research was undertaken to determine whether sex and thyroid hormones influence carcinogenesis and if so at what stages and in which tissues and cells.

### MATERIALS AND METHODS

For these experiments 521 black-hooded, laboratory bred rats aged 2 to 3 months were used. Each rat received under ether-anaesthesia an injection of 0·1 ml. of a saturated acetone solution of 9,10-dimethyl-1,2-benzanthracene (DMBA) into the right and left salivary gland complex. In order to deposit the carcinogen in all 3 salivary glands 0·05 ml. was injected in an anterior direction into the submandibular plus sublingual gland and the other 0·05 ml. in a posterior

* Gibb Senior Fellow, British Empire Cancer Campaign for Research.
† Working with a grant from the British Empire Cancer Campaign for Research.

direction into the parotid. Previous experiments with the injection of equal amounts of acetone (Cherry and Glucksmann, 1965) had failed to induce any tumours and were not repeated. Four to 6 weeks before the injection of DMBA, 122 males and 115 females were castrated surgically under ether anaesthesia.

A total of 112 intact and castrate male and female animals received no treatment additional to the DMBA injection and were allowed to survive until clinical symptoms of tumours appeared. Testosterone propionate (Ciba) in 30 mg. pellets was inserted subcutaneously into 93 intact and castrate males and females. Stilboestrol (B.P.) was added to the drinking water (0·1 mg./1000 ml.) of 83 intact and castrate males and females, making a daily dose of about 0·002 mg. per rat. Another 79 intact and castrate males and females had testosterone pellets inserted and were given stilboestrol in their drinking water. L-Thyroxine sodium (Eltroxin, Glaxo) was added to the drinking water (1 mg./1000 ml.) giving a daily dose of approximately 0·02 mg. for each of 77 intact and castrate males and females. Methylthiouracil (B.D.H., 1 g./1000 ml.) was administered in the drinking water to 77 intact and castrate males and females, i.e. a daily dose of about 0·02 g. per rat.

At autopsy the salivary glands were fixed in Bouin's fluid and the tumours in Zenker-acetic if they could be dissected from the gland complex. In addition the pituitary, adrenals, thyroid and parathyroid, liver, spleen with pancreas, kidneys and the gonads with cervix and uteri or seminal vesicles and prostate were fixed and prepared for histological examination. After paraffin-embedding, sections were cut at 8 $\mu$ and stained with haematoxylin and eosin, the periodic acid-Schiff technique with prior diastase digestion, Southgate's mucicarmine stain, Van Gieson's method or with carmalum-aniline blue-orange G.

### RESULTS

The first carcinoma verified histologically occurred 40 days and the first sarcoma 41 days after the injection of the carcinogen. Some animals died or had to be killed before this period and are not considered " at risk ". The main cause for early death or killing was ulceration of the skin in the region of the salivary glands and of the 521 rats, 69 were killed for this reason, i.e. 13%. In the various treatment groups the incidence of ulceration varied from 6% in animals given additionally methylthiouracil to 23% in those with testosterone pellets. In both intact males and castrate females the incidence of ulceration was 17% while in castrate males it was only 7% and in intact females 11%. There is no correlation between the incidence of ulceration and that of carcinomas or sarcomas nor between ulceration and the incidence of cancers according to treatment groups, to sex or castration. In previous experiments injection was made into one gland complex only either after surgical exposure or through the skin. No definite variation in the incidence of ulceration with the mode of inserting the carcinogen or with uni- or bilateral application could be discerned.

The latest tumours to develop allowed rats to survive for about 600 days after injection of the DMBA. Others survived for up to 769 days after injection without tumours. The animals were killed as soon as firm lumps were felt in the treated region or when the rat appeared to suffer discomfort. Some animals had intercurrent disease such as breast tumours, leukaemias, pituitary tumours and, in the group treated with methylthiouracil, adenomas or carcinomas of the thyroid. Castration caused adrenal hyperplasia, the appearance in the pituitary of castration cells, which persisted in spite of stilboestrol administration, but were eliminated by testosterone treatment of both males and females. Intact and castrate females treated with testosterone ± stilboestrol showed ossification in the clitoris. Epithelial cords and secretory ducts in the thymus occurred with increased frequency after stilboestrol treatment and were decreased by testosterone application (Cherry, Eisenstein and Glucksmann, 1967).

*The effect of sex, castration, of sex and thyroid hormones on the induction of carcinomas*

The results of the 24 experimental variations are illustrated in the histograms of Fig. 1. The highest cancer incidence is seen in intact males and is significantly greater than that in castrate males and in intact and castrate females. The

FIG. 1.—Histograms showing the effect of sex, castration, L-thyroxine, methylthiouracil, testosterone with and without stilboestrol and of stilboestrol on the induction of carcinomas in intact and castrate rats.

differences between these last three groups though striking do not reach the 95% confidence level. Their rate of development decreases in the same order (Fig. 2) and suggests that as regards carcinomas intact males rank highest, followed by castrate males, intact and lastly castrate females. All four groups have a linear relation between increase in percentage and time and though many animals survived for periods longer than 200 days no more carcinomas appeared after that period.

As might be expected from these data, treatment with *testosterone* increases and accelerates the induction of carcinoma in castrate males and in intact and castrate females (Fig. 2). The final percentages are significantly increased in intact and castrate females (Fig. 1), but only slightly in castrate males.

*Stilboestrol* decreases and retards cancer induction significantly in intact males and markedly in castrate males. It does not greatly influence the process in intact and castrate females. As Fig. 1 shows, the general level of cancer induction in the four groups is lowered to almost the level of castrate females and thus contrasts with the effect of testosterone.

FIG. 2.—Graphs illustrating the influence of sex, castration, testosterone with and without stilboestrol and of stilboestrol on the induction of carcinomas. The standard errors are indicated by vertical lines.

The combined treatment with *testosterone and stilboestrol* closely approximates treatment with testosterone only, causing the same high incidence of carcinomas (Fig. 1), though not accelerating the process to the same extent (Fig. 2). These experiments suggest that the incidence and rate of formation of carcinomas increases with the " maleness " of animals.

The effects of treatment with L-*thyroxine* (Fig. 3) or with *methylthiouracil* (Fig. 4) appear to be generally inhibitory on the incidence of carcinomas (Fig. 1), methylthiouracil being more effective than L-thyroxine. The rate of carcinogenesis however, is depressed more by L-thyroxine than by methylthiouracil

FIG. 3.—Graph illustrating the influence of L-thyroxine on the induction of carcinomas in intact and castrate male and female rats. The numerals " 608 " after the arrow indicate that the tumour incidence remained at this level for the period of 608 days after injection. The numerals in Fig. 3, 4, 7, 9, 10, 11 and 12 indicate a steady level of tumour incidence up to the day indicated.

(Fig. 4). Both agents affect intact and castrate females more than intact and castrate males, and indeed the effect on intact males is not significant except for the slowing down of tumour development by L-thyroxine. In intact females and castrate males L-thyroxine prolongs the period of risk of carcinogenesis very significantly (Fig. 3) and in the castrate males changes the cumulative curve from a straight line to a biphasic one. The greatest effect is seen in castrate females with the suppression of carcinomas by methylthiouracil and their reduction by L-thyroxine.

The differences in the reaction of animals with different sexual status to the various agents used is illustrated by Fig. 5. Intact males produce carcinomas in the 50 to 75% range with the exception of rats treated with stilboestrol. In castrate males the range lies between 38 and 56%, again with the exception of stilboestrol-treated animals. In intact females the cancer incidence varies between 12% for methylthiouracil treatment and 72% for testosterone admini-

FIG. 4.—Graph illustrating the influence of methylthiouracil on the induction of carcinomas in intact and castrate male and female rats.

FIG. 5.—Histograms showing the effect of L-thyroxine, methylthiouracil, testosterone with and without stilboestrol and of stilboestrol on the incidence of carcinomas in intact and castrate male and female rats.

stration. In castrate females the variations are even wider: 0% for methylthiouracil, 6% for L-thyroxine, 63% for testosterone and 68% for testosterone plus stilboestrol. It thus seems that "maleness" not only encourages the production of carcinomas, but fixes the responsiveness to DMBA at a high level. Castration increases the pronounced variability in the response of females to hormonal treatments additional to DMBA administration as measured by the induction of carcinomas.

The stage at which sex, sex hormones and thyroid hormones influence the carcinogenic process appears to be linked with the formation of an epithelial cyst and sinus from persisting glandular ducts which undergo squamous metaplasia. The injected carcinogen causes necrosis of parts of the glands, interferes with the autolytic and phagocytic disposal of the necrotic tissue, elicits squamous metaplasia in persisting larger ducts which proceed to encyst the killed gland (Cherry and Glucksmann, 1965). The enlarging cyst filled with debris and with exfoliated keratinised material tends to transform into a sinus which discharges its content through the skin or into the buccal cavity. Carcinogenic material within the cyst induces proliferation of the epithelium and its transformation into carcinomas. Excessive necrosis may occur and lead to early ulceration which inhibits the formation of a sinus though, depending on the duration and extent of the ulcerative process, an attempt at epithelialisation of the debris may start from the epidermis, and give rise to the carcinomas appearing later (Fig. 3). Failure of the persisting ducts to proliferate and to undergo squamous metaplasia is another reason why sinuses do not develop. The incidence and extent of squamous metaplasia differs greatly in the two sexes, being much less in females than in males. In females this reaction of regenerating ducts is further inhibited by L-thyroxine, methylthiouracil and stilboestrol, but markedly increased by testosterone. In males too stilboestrol decreases the incidence and extent of squamous metaplasia. It is noteworthy that squamous metaplasia and with it sinus formation are reduced in all experiments in which the incidence of carcinomas is low. There is also very good correlation within a given experiment between squamous metaplasia, sinus and carcinoma formation which are found in rats with, and are absent in those without tumours.

Sinuses also account for the limitation in time of cancer risks: the toxic effects of the encysted carcinogen-impregnated debris together with the enlargement due to accumulated exfoliated material cause thinning of the sinus epithelium and unless carcinoma formation has supervened, the sinus bursts and its contents come into contact with the surrounding stroma where it may elicit the formation of sarcomas. With the disappearance of the sinus the risk of carcinoma development disappears. The carcinogen can produce carcinomas only if in contact with the cuticular surface of an epithelium, while at its basal surface it comes into contact with mesenchymal elements and elicits sarcomas.

*The effect of sex, castration, of sex and thyroid hormones on the induction of sarcomas*

The final results of the 24 modifications of experimental conditions on the formation of sarcomas are given in the histograms of Fig. 6 and show much less variation than those for carcinoma induction by the same means (Fig. 1). If the cumulative incidence of sarcomas is plotted against time (Fig. 7) differences in rate of development can be discerned though they are not as great as in the

FIG. 6.—Histograms showing the effect of sex, castration, L-thyroxine, methylthiouracil, testosterone with and without stilboestrol and of stilboestrol on the induction of sarcomas in intact and castrate rats. (See Fig. 1 for key).

FIG. 7.—Graph illustrating the influence of sex and castration on the induction of sarcomas.

carcinomas (Fig. 2). Nevertheless sarcoma induction is more rapid in intact males than in intact females and is intermediate in the castrate animals. The cumulative incidence in all 4 groups is biphasic with a steep initial and a more gradual subsequent slope, which extends to about 500 days in the spayed females. The late development of some sarcomas obscures differences between the various experimental groups, which can be shown in the incidence of sarcomas present at 200 days after the injection of the carcinogen (Fig. 8). The percentage of sarcomas is significantly decreased in intact males by treatment with stilboestrol,

FIG. 8.—Histograms showing the effect of sex, castration, L-thyroxine, methylthiouracil, testosterone with and without stilboestrol, and of stilboestrol on the induction of sarcomas at 200 days after injection in intact and castrate rats. (See Fig. 1 for key).

in castrate males by stilboestrol and also by L-thyroxine, in intact females greatly, but not significantly, by L-thyroxine and in spayed females moderately by L-thyroxine and by methylthiouracil. The incidence of sarcomas is increased significantly by testosterone in intact females and by testosterone plus stilboestrol in spayed females.

The rates of sarcoma induction in testosterone and stilboestrol treated rats is shown in Fig. 9. Except for testosterone treated intact males the graphs are again biphasic. The treated animals fall distinctly into 2 groups according to the hormonal treatment as indicated by the arrow. Testosterone accelerates and stilboestrol slows down, but extends in time the development of sarcomas. Treatment with testosterone plus stilboestrol (Fig. 10) closely approximates the effect of testosterone alone. L-thyroxine retards the induction of sarcomas (Fig. 11) as it does that of carcinomas (Fig. 3) and again more in females than in males,

FIG. 9.—Graph illustrating the influence of testosterone and of stilboestrol on the induction of sarcomas in intact and castrate male and female rats. The arrow indicates the gap between testosterone and stilboestrol treated animals.

FIG. 10.—Graph illustrating the influence of testosterone plus stilboestrol on the induction of sarcomas in intact and castrate male and female rats.

FIG. 11.—Graph illustrating the influence of L-thyroxine on the induction of sarcomas in intact and castrate male and female rats.

FIG. 12.—Graph illustrating the influence of methylthiouracil on the induction of sarcomas in intact and castrate male and female rats.

but the inhibition of sarcomas in spayed females does not equal that of carcinomas. In all four groups of animals methylthiouracil (Fig. 12) has only a slight effect on the development of sarcomas and does not inhibit or even prevent sarcoma formation as it does carcinomas in the intact and castrate females (Fig. 3).

The final percentage of induced sarcomas (Fig. 13) has the same range in intact and castrate males and females, i.e. 68 to 84% for intact males with the exception of 52% for stilboestrol treatment, 67 to 95% for castrate males with the exception

FIG. 13.—Histograms showing the effect of L-thyroxine, methylthiouracil, testosterone with and without stilboestrol and of stilboestrol on the incidence of sarcomas in intact and castrate male and female rats.

of 59% for L-thyroxine treatment, 67 to 92% for intact females with the exception of 56% for L-thyroxine treatment and 69 to 100% for castrate females. From these data it might appear that the sensitivity of the stroma to the carcinogen is not affected by hormonal treatment. A similar analysis for sarcoma induction by 200 days (Fig. 14) presents a different picture, however ; as for carcinomas (Fig. 4) the intact males show an effect only of stilboestrol and remarkably constant levels for all other treatments. The variability in response of castrate males is much greater with low levels for stilboestrol and L-thyroxine and with high ones in the testosterone treated and the control groups. Intact females have high incidences in the testosterone, stilboestrol and testosterone plus stilboestrol groups and a low one in the L-thyroxine treated animals. In castrate females the testosterone and testosterone plus stilboestrol treated rats form a high level group and those treated with L-thyroxine and methylthiouracil a low-level one.

FIG. 14.—Histograms showing the effect of L-thyroxine, methylthiouracil, testosterone with and without stilboestrol and of stilboestrol on the incidence of sarcomas at 200 days after injection in intact and castrate male and female rats.

Again the slow rate of development of sarcomas in the later periods obscures differences in effectiveness of treatment.

The modification of sarcoma induction by the various changes in hormonal conditions parallels that of carcinomas. Even for the final incidence the correlation coefficient for carcinomas and sarcomas in the 24 experimental groups is $r = + 0{\cdot}499 \pm 0{\cdot}21$, but is considerably greater for carcinomas and the induction of sarcomas by 200 days (Table I) when $r = + 0{\cdot}666 \pm 0{\cdot}21$.

TABLE I.—*The Influence of Various Hormonal Treatments on the Incidence of Carcinomas and Sarcomas*

| Treatment additional to DMBA | Number at risk | Carcinoma % | Sarcoma present by day 200 % | Sarcoma absent day 200 to 769 % |
|---|---|---|---|---|
| None | 102 | 48 | 70 | 9 |
| Testosterone | 72 | 61 | 84 | 3 |
| Testosterone + Stilboestrol | 72 | 58 | 78 | 6 |
| Stilboestrol | 69 | 28 | 51 | 17 |
| L-Thyroxine | 65 | 31 | 48 | 22 |
| Methylthiouracil | 72 | 26 | 58 | 18 |

The deposits of DMBA are initially more toxic to connective tissue than to the epithelium and while inflammatory and fibroblastic reaction capable of removing the necrotic material and forming a scar is absent, encystation of the debris proceeds with some success. In the later stages fibroblasts and inflammatory cells which are adapted to the toxic effects of DMBA appear and form a capsule and also the sarcomas. Since the animals were killed at the earliest sign of tumour formation and since the early tumours are often carcinomas, it is natural that sarcomas are not found in 100% of the rats at risk. Indeed a 100% incidence of sarcomas is seen only in castrate females given testosterone plus stilboestrol and figures of 95% in castrate males without additional hormonal treatments and in testosterone treated castrate males. The failure to produce sarcomas after the end of the period of risk for carcinomas, i.e. from 200 days onward, varies with the type of treatment. Of the 452 animals at risk 54 or 12% did not have sarcomas in the period from 200 to 769 days after injection. The percentage of negative results was 10% for intact and 13% for castrate males, 11% for intact and 14% for castrate females. In the four control groups sarcomas were absent in 9%, but in only 3% of testosterone and in 6% of testosterone plus stilboestrol treated rats (Table I). In stilboestrol treated animals the failure rate rose to 17% and was 18% for methylthiouracil and 22% for L-thyroxine treated rats. Within the treatment groups the status of the gonads has a striking influence on the development of sarcomas: in intact males given stilboestrol alone or in combination with testosterone 20% of 40 rats did not have sarcomas, against only 6% of 85 rats not so treated. In castrated males treated with thyroxin or with methylthiouracil the failure rate was 32% as compared with 4% in all other animals, and in castrated females the respective figures are 23% and 8%.

In the absence of sarcomas the salivary glands are atrophic and usually enclosed by a fibrotic and thickened capsule. The failure to produce sarcomas may be due to a low sensitivity of the animals to the carcinogen which allows them to form a stable scar and capsule. Alternatively early ulceration may have been successful in discharging all of the carcinogen. We have no direct data about the degree of ulceration in the individual animals which did not form sarcomas later on. On the other hand, as mentioned above, we have information about the number of animals which had to be killed early because of excessive ulceration; if excessive ulceration is the main cause of inhibiting sarcomas, one would expect some correlation between the percentage of animals showing early ulceration and the failure rate for sarcoma production in the various experimental groups. The correlation coefficient for these parameters is $r = -0{\cdot}11 \pm 0{\cdot}21$ which does not support the hypothesis. The variation in the failure to produce sarcomas with treatment groups and the strong inverse correlation with the incidence of carcinomas and of sarcomas up to 200 days (Table I) suggests that the hormonal treatments have altered the sensitivity of the target tissues to the carcinogen.

### DISCUSSION

Quite apart from the differences in the incidence of cancers of accessory sex organs such as the breast in men and women, there are appreciable variations in the sex ratio for cancers of the buccal cavity, pharynx, larynx, oesophagus, stomach, lung and bronchus which are twice to twenty times as frequent in males as in females. Some of these differences can be attributed to environmental factors such as occupational hazards, smoking and drinking which promote carcinogenesis in males and which are reflected also in the variation in the incidence of some cancers in different countries and, within a country, with social status.

There are, however, possibly some factors inherent in the male and the female which promote or inhibit the development of carcinomas at various sites. An understanding of these factors might help us to use them in the prevention or in the therapy of cancers at least as adjuvants to other techniques. The aim of the experiments reported here is to elucidate in animals living in the same environment what factors influence carcinogenesis in males and females. Because of the sex dimorphism in the salivary glands (Lacassagne, 1940) we have chosen this system in the hope of being able to discover sex-linked gradations of reaction to carcinogens.

The most obvious differences between male and female salivary glands are concerned with the volume and secretory activity of the tubules of the submandibular gland which in males greatly exceed those of females and which diminish after castration or hormonal treatments (Arvy *et al.*, 1950 ; Grad and Leblond, 1949 ; Jacoby and Leeson, 1959 ; Lacassagne, 1940*a*, *b* ; Shafer and Muhler, 1956). The male submandibular gland of mice also produces more of the nerve growth factor (Levi-Montalcini, 1965) and of the epidermal growth factor (Cohen, 1965) than the female, though the secretion can be stimulated in the female by testosterone. Irradiation injures the acini and induces regeneration from the secretory tubules of the submandibular and the intercalated ducts of the sublingual glands which later result in the appearance of adenomas, and both these processes are more marked in males than in females. With locally applied carcinogens Steiner (1942) and Bauer and Byrne (1950) failed to find any sex difference in carcinogenesis, though with feeding of carcinogens Heiman and Meisel (1946) and Reuber (1960) did find an effect of sex. The present series of experiments demonstrates a sex difference in the induction of carcinomas and to a lesser extent also of sarcomas, but these differences are not linked with the morphological dimorphism of the submandibular. In fact all three glands react in the same manner and the variation with sex in tumour incidence concerns sarcomas as well as carcinomas, a fact not obvious in our previously reported experiments (Cherry and Glucksmann, 1965), in which, at least in the females only the left side of the glandular complex was injected with the carcinogen and comparison between the two sexes was based on this procedure. In the present series consistently fewer sarcomas are induced and more slowly in females than in males and testosterone promotes and accelerates, while stilboestrol inhibits and retards the formation of these tumours. There is also a significant correlation between carcinoma and sarcoma induction in these experiments, particularly if the first 200 days after injection are considered. In the previously recorded experiments the rate of sarcoma formation in females was slower than that in males (cf. Fig. 21, Cherry and Glucksmann, 1965) but in the absence of confirmatory evidence the difference was not considered significant at that time. Subsequent results have established this difference as consistent and real. The sex difference in the induction of carcinomas is greater and always consistent.

For carcinomas the influence of sex and sex hormones is decisive at the stage of sinus formation and encystation of the necrotic glandular mass that contains and retains the DMBA as crystals. In the larger ducts of all three major glands proliferation of the epithelium with squamous metaplasia is one of the conditions for encystation, and is greater in males than females and is promoted by testosterone and inhibited by stilboestrol. It is tempting to link the greater capacity for epithelial regeneration in males with the larger production of the epidermal growth factor in the submandibular gland and thus ultimately with the higher incidence of carcinomas. There is no evidence that the same factors are responsible for the more frequent and rapid proliferation of adapted fibroblasts leading to the appearance of sarcomas. These sex and hormonal effects are modifying only and the capacity for these responses is present in males, females and castrates. Indeed as Fig. 2 suggests, stilboestrol accelerates slightly the induction of carcinomas in castrate females, though the difference is not statistically significant.

A stimulating effect on the development of cervico-vaginal sarcomas by testosterone and absence of such promotion by oestrogens is seen after application of DMBA to the female genital tract (Cherry and Glucksmann, 1960). Castration retards and very greatly reduces the incidence of such tumours and neither stilboestrol nor oestradiol, which stimulate the growth of the vaginal, cervical and uterine stroma, accelerate and increase the development of sarcomas. Testosterone, on the other hand, which fails to stimulate the growth of the normal stroma of the female genital tract in castrate animals, promotes and increases the production of sarcomas in the castrate rat, though it retards it in the intact animal. Furthermore, while oestradiol and stilboestrol induce proliferation and squamous metaplasia in the cervico-vaginal epithelium, they inhibit both these processes in the ducts of salivary glands after DMBA-injection. Thus it seems that the promoting action of testosterone on carcinogenesis and the lack of growth stimulation by stilboestrol in a DMBA-treated tissue may differ from their normal role in the reproductive context. It should be remembered, however, that though the doses of the hormones given are not greatly beyond the physiological level, the long-term continuous application may well produce unphysiological conditions and these may be reinforced by castration. In any case these experiments on animals kept under the same environmental conditions establish clearly that sex and sex hormones modify the cancer incidence and make it likely that inherent differences in combination with environmental factors may be responsible for the marked sex differences in human carcinomas.

That castration may add to the unphysiological conditions for hormonal actions is illustrated clearly by the experiments with L-thyroxine and methylthiouracil. Thyroidectomy affects the growth of the submaxillary gland in females more than in males (Hammett, 1923). Methylthiouracil has little effect on the rate of development of carcinomas in males, but reduces it in intact females and suppresses it in the castrate females. L-Thyroxine has little influence on the induction of carcinomas in intact males, slows it down in castrate males and even more so in intact females and almost suppresses it in castrate females. The effects of L-thyroxine and methylthiouracil on the incidence of sarcomas differ from that on carcinomas in that methylthiouracil has no appreciable influence on sarcomas and L-thyroxine slows down the process almost equally in all four groups. In the female genital tract treated with DMBA (unpublished results) L-thyroxine as well as methylthiouracil accelerate and increase the production of cervico-vaginal sarcomas in castrates, but inhibit and retard the induction of vulval carcinomas. In intact rats the production of sarcomas is not greatly affected by the application of either L-thyroxine or methylthiouracil. It is surprising that as regards carcinogenesis, L-thyroxine and methylthiouracil should have a similar action when their general effects on metabolism, on the growth and secretion of the submaxillary gland (Arvy and Gabe, 1950*a* ; Shafer and Muhler, 1956), on the thyroid and on the pituitary are diametrically opposed.

As with the sex hormones, the action of thyroid hormones on carcinogenesis appears to be different from that in their normal context. Thus the synergistic action of thyroxine and testosterone (Grad and Leblond, 1949) and the antagonistic action of thyroxine and oestrogens (Arvy and Gabe, 1950*b*) on the increase in size and secretory activity of the submaxillary gland are not simply paralleled by the effect on the development of tumours. Here again the long continued administration of the substances may be responsible for unphysiological conditions in the animal and as the differences in the efficacy of the agents in intact and castrate males and females show, castration adds to the abnormal status and modifies the effects. It is probable that the difference in action of the sex and thyroid hormones on tissues undergoing carcinogenesis is due to some general metabolic rather than to a specific endocrine process.

### SUMMARY

The right and left salivary gland complex of rats was injected with DMBA and the effect of sex, castration and of the treatment with testosterone, stilboestrol, testosterone plus stilboestrol, L-thyroxine and methylthiouracil on the induction of carcinomas and sarcomas in male and female animals was studied.

The first carcinomas and sarcomas appeared after 40 days, the last carcinomas before 200 days and the last sarcomas at about 500 days. The cumulative percentage of carcinomas rose linearly with time, but that of sarcomas was biphasic with an initial steep and a subsequent gradual slope.

More carcinomas were elicited more rapidly in males than in females and castrated rats. The same applied to the induction of sarcomas particularly up to 200 days, but in later stages the sex difference in incidence was obscured by the tardy development of tumours.

Testosterone increased and accelerated the induction of carcinomas and sarcomas in females and castrates while stilboestrol decreased and retarded tumour development in all but intact female rats. Testosterone and stilboestrol given simultaneously had an effect similar to testosterone alone.

L-Thyroxine slowed the development of carcinomas and sarcomas in intact and castrate males and females and also lowered significantly the percentage of carcinomas induced in intact and castrate females.

Methylthiouracil decreased the incidence of carcinomas in intact and even more in castrate females, but had no significant effect on the rate of carcinogenesis in males and on the development of sarcomas in both groups of females.

The authors are grateful to Professor Dame Honor B. Fell, F.R.S. for reading the manuscript.

### REFERENCES

Arvy, L., Debray, Ch. and Gabe, M.—(1950) *C.r. Séanc. Soc. Biol.*, **144**, 111.

Arvy, L. and Gabe, M.—(1950*a*) *C.r. hebd. Séanc. Acad. Sci., Paris*, **230**, 1611.—(1950*b*) *C.r. hebd. Séanc. Acad. Sci., Paris*, **230**, 2333.

Bauer, W. H. and Byrne, J. J.—(1950) *Cancer Res.*, **10**, 755.

Cherry, C. P., Eisenstein, R. and Glucksmann, A.—(1967) *Br. J. exp. Path.*, **48**, 90.

Cherry, C. P. and Glucksmann, A.—(1960) *Br. J. Cancer*, **14**, 489.—(1965) *Br. J. Cancer*, **19**, 787.

Cohen, S.—(1965) *Devl. Biol.*, **12**, 394.

GLUCKSMANN, A. AND CHERRY, C. P. —(1962) *Radiat. Res.*, **17**, 186.
GRAD, B. AND LEBLOND, C. P.—(1949) *Endocrinology*, **45**, 250.
HAMMETT, F. S.—(1923) *Am. J. Anat.*, **32**, 75.
HEIMAN, J. AND MEISEL, D.—(1946) *Cancer Res.*, **6**, 617.
JACOBY, F. AND LEESON, C. R.—(1959) *J. Anat.*, **93**, 201.
JUNQUEIRA, L. C. U., FAJER, A. B., RABINOVITCH, M. AND FRANKENTHAL, L.—(1949) *J. cell. comp. Physiol.*, **34**, 129.
LACASSAGNE, A.—(1940*a*) *C.r. Séanc. Soc. Biol.*, **133**, 180.—(1940*b*) *C. r. Séanc. Soc. Biol.*, **133**, 539.
LEVI-MONTALCINI, R.—(1965) *Archs Biol., Liege*, **76**, 219.
REUBER, M. G.—(1960) *J. natn. Cancer Inst.*, **25**, 1141.
SHAFER, W. G. AND MUHLER, J. C.—(1953) *J. dent. Res.*, **32**, 262.—(1956) *J. dent. Res.*, **35**, 922.
STEINER, P. E.—(1942) *Archs Path.*, **34**, 613.